# Genomic BCR-ABL1 breakpoint characterization by a multi-strategy approach for “personalized monitoring” of residual disease in chronic myeloid leukemia patients

**DOI:** 10.18632/oncotarget.23971

**Published:** 2018-01-05

**Authors:** Cosimo Cumbo, Luciana Impera, Crescenzio Francesco Minervini, Paola Orsini, Luisa Anelli, Antonella Zagaria, Nicoletta Coccaro, Giuseppina Tota, Angela Minervini, Paola Casieri, Claudia Brunetti, Antonella Russo Rossi, Elisa Parciante, Giorgina Specchia, Francesco Albano

**Affiliations:** ^1^ Department of Emergency and Organ Transplantation, Hematology Section, University of Bari, 70124 Bari, Italy

**Keywords:** genomic BCR-ABL1 breakpoint, chronic myeloid leukemia, FISH, MinION sequencing, droplet digital PCR

## Abstract

For monitoring minimal residual disease (MRD) in chronic myeloid leukemia (CML) the most recommended method is quantitative RT-PCR (RT-qPCR) for measuring BCR-ABL1 transcripts. Several studies reported that a DNA-based assay enhances the sensitivity of detection of the BCR-ABL1 genomic rearrangement, even if its characterization results difficult. We developed a DNA-based method for detecting and quantifying residual BCR-ABL1 positive leukemic stem cells in CML patients. We propose two alternative approaches: the first one is a fluorescence *in situ* hybridization (FISH)-based step followed by Sanger sequencing; the second one employs MinION, a single molecule sequencer based on nanopore technology. Finally, after defining the BCR-ABL1 genomic junction, we performed the target CML patient–specific quantification, using droplet digital PCR (ddPCR). FISH and MinION steps, respectively, together with ddPCR analysis, greatly reduce the complexity that has impeded the use of “personalized monitoring” of CML in clinical practice. Our report suggests a feasible pipeline, in terms of costs and reproducibility, aimed at characterizing and quantifying the genomic BCR-ABL1 rearrangement during MRD monitoring in CML patients.

## INTRODUCTION

For monitoring minimal residual disease (MRD) in chronic myeloid leukemia (CML) the most recommended method is quantitative RT-PCR (RT-qPCR) for measuring BCR-ABL1 transcripts [1]. In the tyrosine kinase inhibitors (TKIs) treatment-free remission (TFR) era, more reliable methods are needed for identifying those CML patients candidates for TFR with the lowest likelihood of relapse. In fact, the probability of relapse after TKIs withdrawal is likely related to the persistence of residual BCR-ABL1 positive (BCR-ABL1+) leukemic stem cells (LSCs), which may be transcriptionally quiescent and TKI-resistant [2], at levels below the threshold of RT-qPCR detection (estimated to be around 10^–5^) [3]. Several studies reported that a DNA-based assay enhances the sensitivity of detection of the BCR-ABL1 rearrangement [4–8]. All these studies demonstrated that using the DNA sequence spanning the BCR and ABL1 gene breakpoints as the target for CML monitoring has several advantages: genomic DNA is more stable, the BCR-ABL1+ cell number is directly measured, and laboratory standardization is simpler. Moreover, an improved sensitivity of a DNA-based qPCR approach compared to that of an RNA-based assay has been reported, increasing the limit of detection up to 10^–7^ [6]. However, the development of a DNA-based assay have to address two main issues: i) the identification of the BCR-ABL1 genomic fusion junctions may be very challenging, due to the characteristics of the genomic area, rich in repetitive elements [9], and to the technical approach based on multiplexed PCR sessions [8]; ii) the need for a standard curve for DNA-based patient-specific probe assays on a qPCR. Nowadays, the first issue has been overcome by next generation sequencing (NGS) analysis, but this technology is still very expensive and not yet within the reach of all laboratories; the second issue can be overcome by the use of a digital PCR platform, which provides absolute molecular quantification without the need for a standard curve. In light of these considerations we developed a DNA-based method for detecting and quantifying BCR-ABL1+ cells in CML patients that can be adopted by laboratories that do not have the resources available to invest in the main NGS platforms currently available on the market. For this purpose, we propose two alternative approaches: the first one is a fluorescence *in situ* hybridization (FISH)-based step that allows simplification of the complex multiplex PCR process; the second one employs MinION, a single molecule sequencer based on nanopore technology [10]. Finally, after defining the BCR-ABL1 genomic junction, we performed the target CML patient–specific quantification, using droplet digital PCR (ddPCR).

## RESULTS

### BCR-ABL1 breakpoint identification

**Figure 1:**
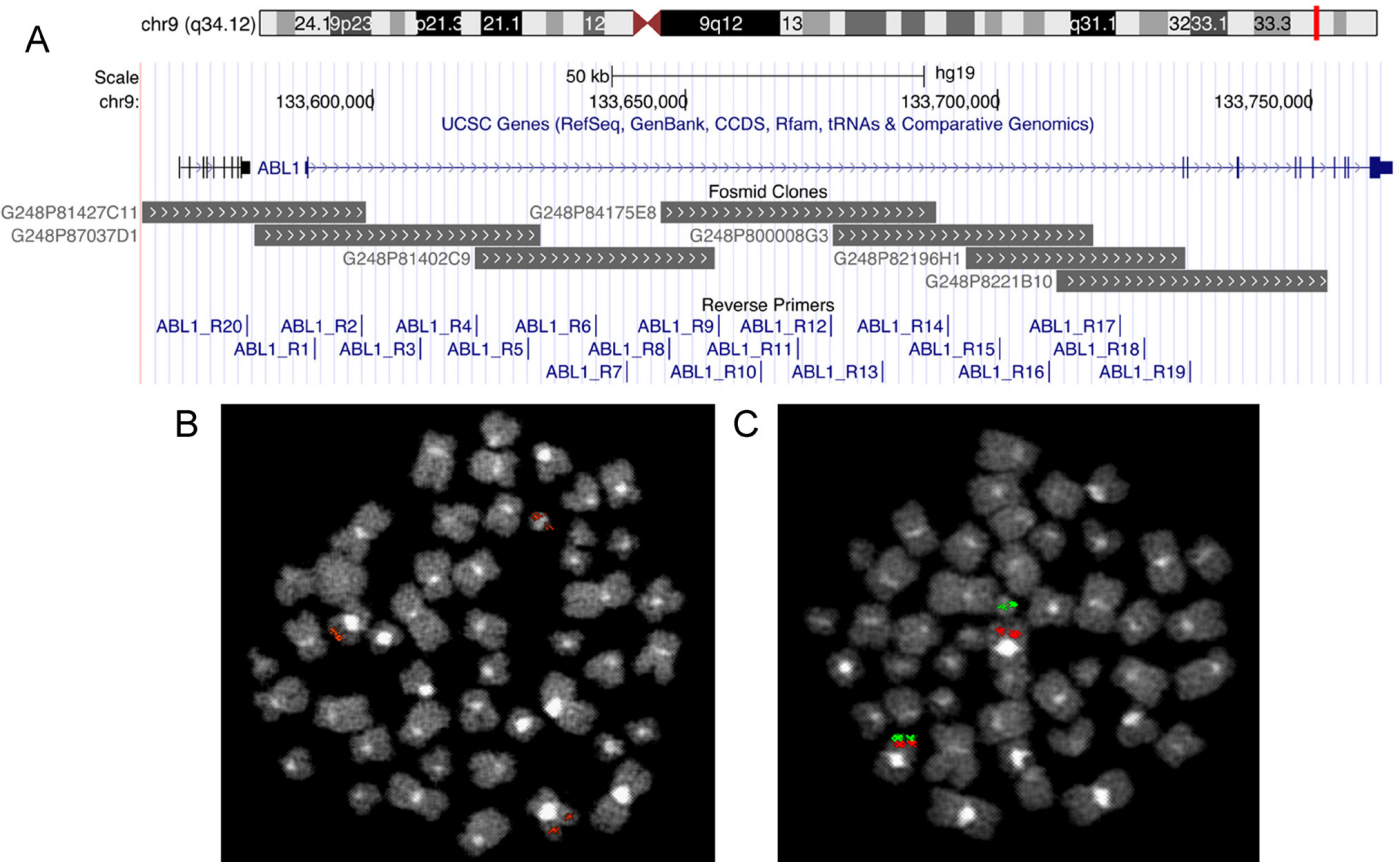
Fosmid clones used for FISH experiments and reverse primers spanning the first intron of ABL1 visualized in UCSC Genome Browser (**A**). Two examples of FISH patterns observed: the splitting of a single fosmid clone (**B**) and of two overlapping fosmid clones (**C**) on der(9) and Ph chromosomes.

To narrow the genomic breakpoint area of analysis in the first intron of gene ABL1, FISH assays were preliminarily performed using seven overlapping fosmid clones. These experiments showed two different FISH patterns (see Table 1 and Figure 1): in 8/10 (80%) CML patients the splitting of two overlapping fosmid clones was observed on the der(9) and Ph chromosomes, in the remaining 2/10 (20%) the FISH pattern showed the splitting of a single fosmid clone on the der(9) and Ph chromosomes. The breakpoint site of gene ABL1 was localized in 3/10 (30%) patients between fosmid clones G248P81427C11 and G248P87037D1, in 3/10 (30%) between G248P84175E8 and G248P800008G3, in 2/10 (20%) between G248P82196H1 and G248P8221B10, in 1/10 (10%) in G248P82196H1, and in 1/10 (10%) in G248P8221B10. The FISH pattern allowed us to reduce to three the number of ABL1 reverse primers to use in the three subsequent long-template PCR experiments; only in one of them, as expected, a PCR product was visualized, that yielded the breakpoint genomic sequence then analyzed by Sanger sequencing (SS).

**Table 1:** Main features of CML patients

Case	Age	Gender	Transcript	FISH pattern		Primers selected	Breakpoint sequence^b^ (5′->3′)
#1	47	M	b3a2	G248P84175E8	9 + der(9)	BCR_F14 ABL1_R13^a^-14-15	GCATCTCCTCCCGGGTCCTGTCTGTGAGCAATACAGCGTGACACCCTACGCTGCCCCGTGGTCCCGGGCTTGTCTCTCCTTGCCTCCCTGTTACCTTTCTgcgtggtggtgggcgcctgtagtaccagctacctgggagcctgaggcagaaaaacggcgtgaacccaggaggcggatctcggcagtgagctcacaccagt
				G248P800008G3	9 + Ph		
#2	89	F	b3a2	G248P82196H1	9 + der(9) + Ph	BCR_F14 ABL1_R15-16^a^-17	GTGGCCTCTGCCCTCTCCCCTAGCCTGTCTCAGATCCTGGGAGCTGGTGAGCTGCCCCCTGCAGGTGGATCGAGTAATTGCAGGGGTTTGGCAAGGACTTaattttctgccattttacaaagttcaagactttcctaccttcctacctcctggctgtgtgaacttggacagaatacttccccctctccctccagtcagtt
#3	90	F	b2a2	G248P81427C11	9 + der(9)	BCR_F13 ABL1_R1^a^-2-3	GCTGCTGGGTGGTTGAGGAGATGCACGGCTTCTGTTCCTAGTCACAAGGCTGCAGCAGACGCTCCTCAGATGCTCTGTGCCTTGGATCTGGCCCCACTCCgtaggacttgaaaatactcactttggagccatgtgggaaaaatcaagtggggaagcagcattccttgtgaattttagatagacagcttctgtcttacctt
				G248P87037D1	9 + Ph		
#4	38	F	b3a2	G248P8221B10	9 + der(9)+ Ph	BCR_F14 ABL1_R17-18-19^a^	GAGTGTGGGGTCCAAGCCAGGAGGGCTGTCAGCAGTGCACCTTCACCCCACAGCAGAGCAGATTTGGCTGCTCTGTCGAGCTGGATGGATACTACTTTTTataattcagaatcagtcccacccctgagatggtattattacccaggaaagaatgcgtgaggatcctctaaatccatagagaaggaaaactaaaacaattt
#5	76	M	b2a2	G248P84175E8	9 + der(9)	BCR_F13 ABL1_R12-13-14^a^	GAACCTACTACTTAACTCCAGAACTCTTTTCCTACAGACTAAGAATACAATCTCAACTAGAAAACTCTAATTCGGTTTTACCACATCCTGACTACTACAGtgattggagtactaagaagagttgtattagtgaaggttcttgagagagagagagagagagagagagagagagagagagtgtgtgtgtgtgtgtgtgtgtg
				G248P800008G3	9 + Ph		
#6	79	M	b3a2	G248P81427C11	9 + der(9)	BCR_F14 ABL1_R1-2^a^-3	ACAACTGCTTGGGAGGCTGAGGGAAGAGAATCGCTTGAACCCAGGAGGCGGAGGTTGCAGTGAGCCGAGCTTGTGCCACTGCATTCCAGCCTGGGCGACAttgggttgcaaactgaactagccacttttttcatggactgccatttttacttgaaactatgacaaactatggttattcagactaaaaagtgtatgaagga
				G248P87037D1	9 + Ph		
#7	64	M	b3a2	G248P82196H1	9 + der(9)	BCR_F14 ABL1_R17-18^a^-19	GGAGTGGCCTCTGCCCTCTCCCCTAGCCTGTCTCAGATCCTGGGAGCTGGTGAGCTGCCCCCTGCAGGTGGATCGAGTAATTGCAGGGGTTTGGCAAGGAagaaaggattatttttatataaaacgatctttcaattttactttaaagacccaaaccattttcttagaatactgtctaaacaagttaatcatgcacagat
				G248P8221B10	9 + Ph		
#8	63	F	b3a2	G248P82196H1	9 + der(9)	BCR_F14 ABL1_R17^a^-18-19	TTCCTGTGCCCCACAGTGGCCTGGAGTCCCCTTTGCCTTAACTCTTTGCCCCATAGTACAGCGGGGTCTGCTCTGATTGTAGGGGCTTCCCACATCCCCCgtcgcccagactggagtgcagttgcacgatctcagctcactgcaagctccgcctcctgggttcacgccattctcctgcctcagcctccctagtagagggt
				G248P8221B10	9 + Ph		
#9	41	M	b2a2	G248P81427C11	9 + der(9)	BCR_F13 ABL1_R1^a^-2-3	CCATGACACTGGCTTACCTTGTGCCAGGCAGATGGCAGCCACACAGTGTCCACCGGATGGTTGATTTTGAAGCAGAGTTAGCTTGTCACCTGCCTCCCTTatacagtgaaacctcgtctttaccaaaaatacaaaaattagccgggtgtggtggcacaagcctgtatgtagtcagctactcaggaggctgaggcatgaga
				G248P87037D1	9 + Ph		
#10	45	M	b2a2	G248P84175E8	9 + der(9)	BCR_F13 ABL1_R12-13-14^a^	CAGGGAGGGCAGGCAGCTAGCCTGAAGGCTGATCCCCCCTTCCTGTTAGCACTTTTGATGGGACTAGTGGACTTTGGTTCAGAAGGAAGAGCTATGCTTGaaaggaagaatttattaccagtagatatgcagtacaagaaatatcaaaggatgtaattaaagcagaaagagaatgataactggtagaaaactggagccac
				G248P800008G3	9 + Ph		

^a^Reverse primer that gave a PCR product.

^b^In upper case the portion of BCR gene, in lower case the portion of ABL1 gene.

Target enrichment for MinION sequencing (MS) consisted in two multiplex long template PCRs. The amplicon obtained in one of them was then barcoded and loaded on MinION for the sequencing run that lasted 24h with 1059 active pores. Sequencing produced a total of 45838 fast5 files containing raw electric signals. Fast5 files were then used as input in Metrichor for base calling and demultiplexing. On the total reads produced, 21465 passed 2D filters and had a recognizable barcode. Mapping results showed a mean sequencing depth, calculated on the BCR gene region retained in the translocation, of around 400X and never below 50X. BAM files were used to calculate the general error rate, which resulted around 8%. Results from SS and MS showed concordance in all the CML cases included in the study.

### Droplet digital PCR breakpoint detection assay

**Figure 2:**
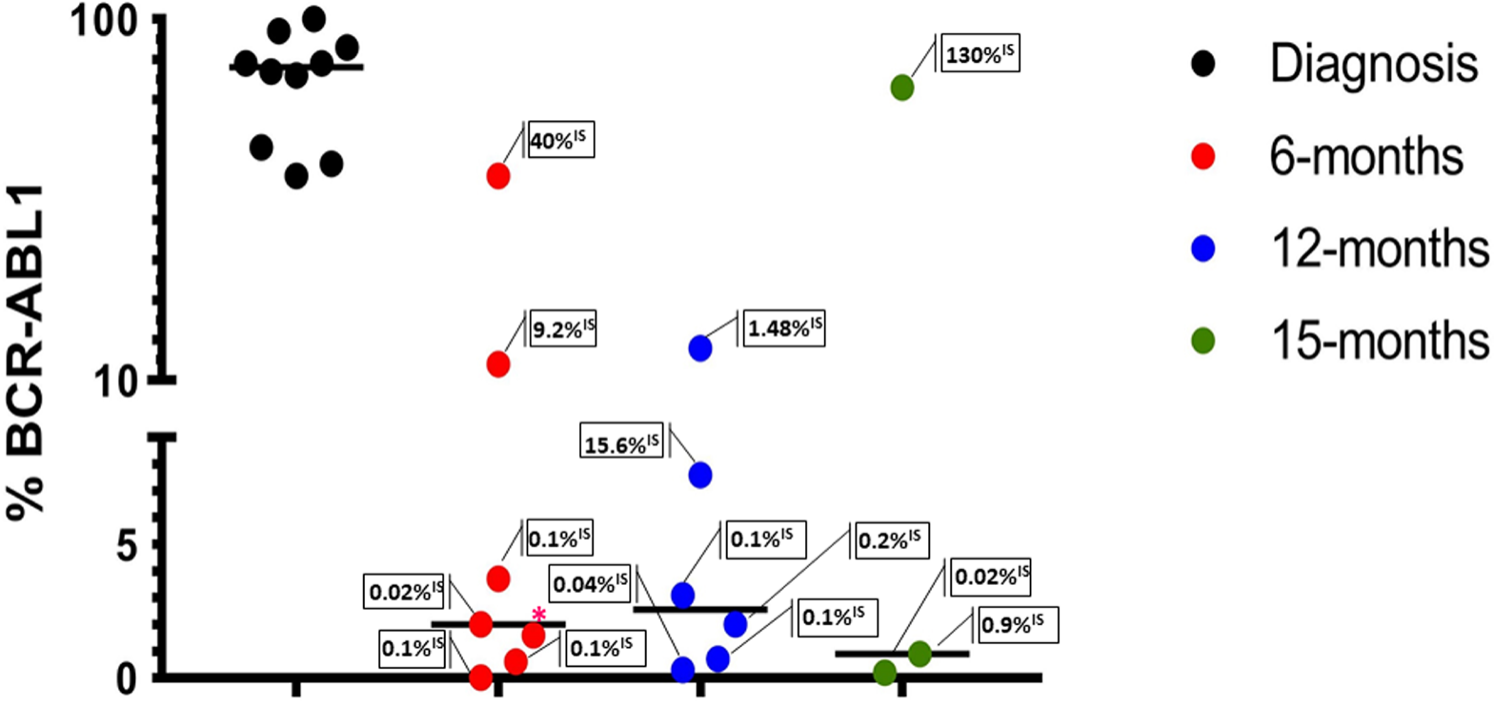
Absolute quantification of BCR-ABL1 genomic breakpoint by ddPCR EG assays at diagnosis and during the follow-up (6-12-15 months) Each dot represents an evaluation; in the boxes the transcript amounts (RT-qPCR) are reported. The lines indicate the median for each group. BCR-ABL1% = [BCR–ABL1/(ZP3/2)] × 100. *%^IS^ not available.

Absolute quantification of the BCR-ABL1 genomic breakpoint was conducted with an EvaGreen (EG) assay. At diagnosis, the patients showed a median leukemic cells abundance of 87% (genomes with the BCR-ABL1 fusion gene) (Figure 2). Sixteen samples corresponding to 6, 12 and 15 months from the CML diagnosis were also evaluated in ddPCR (Figure 2). At all these follow-up points, except one, results were comparable to RT-qPCR results. In our series a percent value of BCR-ABL1 rearranged cells less than four detected by ddPCR genomic analysis, corresponded in most cases (80%) to ≤0.1%^IS^ revealed by the RT-qPCR test; moreover, all CML cases with a ddPCR percent value of BCR-ABL1 rearranged cells more than seven showed a transcript amount corresponding to >1%^IS^ (Figure 2).

**Figure 3:**
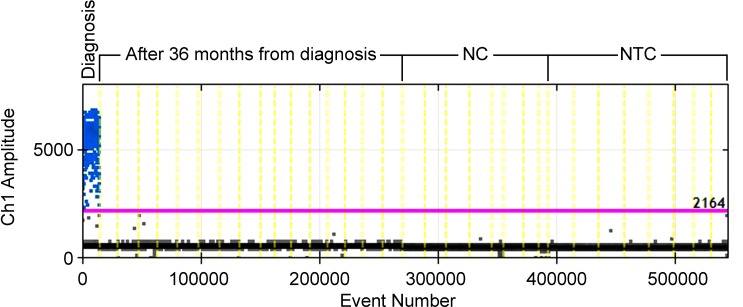
ddPCR TM assay for case#1 at diagnosis and 36 months after diagnosis NC, Negative Control. NTC, No Template Control

We tested case #1 by EG assay also at the time when molecular response (MR) 4.5 was reached [1, 3, 14], after 36 months from diagnosis: the ddPCR analysis did not show positive droplets. Therefore, to increase the specificity, the depth of analysis and the signal-to-noise ratio, a TaqMan (TM) hydrolysis probe assay was designed for this case. By the TM assay the quantification of BCR-ABL1 genomic breakpoint was conducted on a total of 120,000 genomes (16 wells containing 7500 genomes each) and confirmed the absence of leukemic cells previously observed in the EG assay. The analysis specificity was confirmed by testing each assay on a negative control (NC) (a CML patient with a different genomic breakpoint) and a no template control (NTC) (Figure 3); no false positive droplets were detected in the controls and a markedly lower background was observed compared to the EG assay.

## DISCUSSION

To date, BCR-ABL1 genomic breakpoint detection and quantification has not been implemented in routine molecular monitoring in CML patients. This circumstance depends mainly on the fact that the technical implementation of this procedure is particularly complex. The advent of targeted high-throughput sequencing has greatly facilitated the entire procedure, but it is only an opportunity for laboratories that can afford a major investment in this type of technology. In our report we propose two technical solutions for BCR-ABL1 genomic breakpoint characterization. The first is the introduction of FISH before the multiplex PCR experiments, thus reducing the number of reverse primers, specific for intron 1 of the ABL1 gene (from 20 to 3), to be used in the experiment, thus simplifying the characterization process of the genomic breakpoint. In this context, the introduction of FISH may appear to be a complication of the analysis, but in fact it must be considered that in a laboratory specialized in CML MRD monitoring, the FISH technique is generally already adopted. The second improvement in the characterization of the BCR-ABL1 genomic breakpoint suggested by our approach is the introduction of nanopore-based sequencing technology. The MinION approach has already been successfully used by our group to develop a diagnostic assay for hematological malignancies [17–18]. MinION works connected to a laptop through a USB3.0 interface; it is able to connect two strands of DNA molecules by a hairpin, and sequence them consecutively [10]. In our hands, the sequencing results produced by MinION were confirmed in all tested CML cases by SS analysis. Moreover, the very low costs, the ease of use, and the length of the reads (hundreds of kilobases), make MinION an ideal tool for target sequencing. Droplet digital PCR analysis provides a more direct measurement of target copy numbers and offers a greater precision and reproducibility, it is easy to perform and does not require replicate analysis or the generation of standard curves for target quantification. Moreover, studies focused on the use of ddPCR in hematological malignancies have demonstrated a more precise quantitation in comparison with RT-qPCR [19–21]. A recent paper described the validation of a personalized DNA-based digital PCR approach for quantifying very low levels of residual disease, which involves the rapid identification of BCR-ABL1 fusion junctions using targeted NGS [22]. The authors of this work concluded that this approach for detecting residual disease was more sensitive than RT-qPCR analysis. In our report the approach was similar but very different in terms of NGS and ddPCR platforms. The strategies here reported demonstrate clearly that the study of MRD in CML by the BCR-ABL1 DNA-based patient-specific probe is feasible: FISH and MinION steps, respectively, together with ddPCR analysis, greatly reduce the complexity that has impeded the use of “personalized monitoring” of CML in clinical practice. Moreover, the use of FISH and MinION in our proposal pipeline markedly reduces the complexity of the procedure and the costs, respectively, compared to the attempts previously proposed [4–8, 22]. At diagnosis, in 5–10% of CML patients the BCR-ABL1 rearrangement is derived from variant translocations other than the standard t(9;22) or from insertion mechanism. In our series, case #10 showed a variant t(4;9;22) rearrangement. This circumstance did not represent a technical problem for our genomic breakpoint characterization approach, as also expected [23].

The ability to study the cellular burden rather than the transcript load may answer questions and so improve our understanding of CML biology. For example, does the burden of BCR-ABL1+ cells at diagnosis have a prognostic relevance? Among all the CML, are there cases that, for the same load of cells with the genomic rearrangement, produce more or less BCR-ABL1 mRNAs? May this occurrence have a prognostic meaning? Definitely, the possibility of establishing the relationship between the cellular burden and the transcript load could better clarify the mechanisms that regulate the transcription clearance kinetic during TKIs treatment, allowing the early detection of CML patients who will not achieve an optimal treatment response at the third month of therapy (i.e. BCR-ABL1^IS^ ≥10%).

Experimental data indicate that CML LSCs are resistant to TKIs despite BCR-ABL1 inhibition (BCR-ABL1 independent resistance) [24–25], suggesting that targeting critical signaling pathways along with BCR-ABL1 will be needed to eliminate MRD. The aim of a quantitative analysis based on DNA-specific patient probes may obviously also be that of CML patient selection as candidates for TFR. In fact, it could be hypothesized that CML patients with a greater number of LSCs in the bone marrow at the time of a deep MR (4.0–4.5), and not detectable by RT-qPCR, may also be those in which molecular relapse occurred within 6–12 months of TFR [26–27]. In conclusion, our report suggests a feasible pipeline, in terms of costs and reproducibility, aimed at characterizing and quantifying the genomic BCR-ABL1 rearrangement during MRD monitoring in CML patients.

## MATERIALS AND METHODS

### Patients

Ten CML patients at diagnosis were included in this study (Table 1). In all cases the presence of the t(9;22) (q34;q11) translocation was verified by conventional cytogenetics and FISH analysis on bone marrow (BM) samples, as previously reported [11–12]. Case #10 showed a complex variant translocation t(4;9;22) (q21;q34;q11). A qualitative RT-PCR for BCR-ABL1 transcript was routinely performed at diagnosis according to the BIOMED-1 Concerted Action protocol [13]. Molecular monitoring and response was assessed by RT-qPCR according to ELN and EUTOS recommendations [1, 3, 14].

### BCR-ABL1 breakpoint identification

#### FISH analysis

With the aim of reducing the number of ABL-specific primers to be used in subsequent multiplex PCR experiments, FISH preliminary analyses were performed on BM samples using seven fosmid clones (G248P81427C11, G248P87037D1, G248P81402C9, G248P84175E8, G248P800008G3, G248P82196H1, G248P8221B10) overlapping the first ABL1 intron (Figure 1) according to the University of California (Santa Cruz, CA, USA) database (http://genome.ucsc.edu/; February 2009 release). Chromosome preparations were hybridized *in situ* with probes labeled by nick translation, as previously reported [11–12].

### Long-template PCR and SS

Genomic DNA (gDNA) was extracted from peripheral blood (PB) using the QIAamp DNA Blood Mini Kit (Qiagen) and quantified with Qubit 2.0 Fluorometer (Life Technologies). For each patient, to amplify the BCR-ABL1 breakpoint region, three long-template PCRs were performed using PrimeSTAR GXL DNA Polymerase (Takara Bio Inc.), a forward primer (BCR13_F for patients with b2a2 transcript, BCR14_F for patients with b3a2 transcript), a reverse primer from a panel of 20 reverse primers spanning the first intron of ABL1 (Figure 1) as previously reported [8], 500 ng of gDNA in a final volume of 50 μl. Thermal-cycling conditions were 98° C for 10 s, 60° C for 15 s, 68° C for 10 min (30 cycles) and 4° C hold. The PCR products were visualized on a 1% agarose-gel, purified using the QIAquick PCR Purification Kit (Qiagen) and analyzed by SS using nested primers (see Supplementary Table 1) to detect the patient-specific BCR-ABL1 genomic junction.

### BCR-ABL1 breakpoint identification by MS

#### Multiplex long-template PCR

For each patient, two multiplex long-template PCRs were performed using the PrimeSTAR GXL DNA Polymerase (Takara Bio Inc.), a forward primer (BCR13_F) and two pools of reverse primers (pool#1: ABL1_R1-R10 and pool#2: ABL1_R11-R20, one for each multiplex PCR) [8], 500 ng of gDNA, in a final volume of 50 μl. Thermal-cycling conditions were 98° C for 10 s, 68° C for 15 min (30 cycles) and 4° C hold. The PCR products were loaded on an 1% agarose-gel. Only in one of the two multiplex PCR for each patient, an amplicon was visualized, that was purified using the QIAquick PCR Purification Kit (Qiagen). Before starting library preparation, we quantified and estimated the purity of samples (Nanodrop).

### Library preparation and MS

According to the 2D Native barcoding genomic DNA (SQK-LSK 208) protocol, the amplicons were end-prepared using the NEBNext Ultra II End Repair/dA-Tailing Module (New England Biolabs Inc.) and barcoded with the ligation of nanopore-specific Native Barcodes (NB01-NB10) using Blunt/TA Ligase Master Mix (New England Biolabs Inc.). Equimolar amounts of each barcoded amplicon were then pulled and 700 ng of the pool were diluted to 58 μl in nuclease-free water and prepared for sequencing with the ligation of Native Barcoding adapters and the Tether using the NEBNext Quick Ligation Module (New England Biolabs Inc.). All purifications were performed with Agencourt AMPure XP beads (Beckman Coulter Inc.). Dynabeads MyOne Streptavidin C1 (Thermo Fisher Scientific) were used to elute the library in the pre-sequencing Mix. After the Platform QC run and the priming of the flowcell, the sequencing mix (37.5 μl of Running Buffer with Fuel Mix, 25.5 μl of Library Loading Bead Kit, 12 μl of the Pre-sequencing Mix) was loaded and the MAP_48Hr_Sequencing_Run.py protocol was started (MinIONflowcell: FLO-MAP106).

### Data analysis

Poretools toolkit was used to extract FASTQ files from FAST5 files. Reads were then aligned on the GRCh37 human reference genome with the BWA-MEM method using specific Nanopore platform parameters with the optional SAM attributes enabled. The SAM files were used for structural variation analysis using the Sniffles tool [15]. The VCF files from Sniffles were finally used with SplitThreader (http://splitthreader.com) software to visualize and detect breakpoints. Error and coverage analyses were conducted using Qualimap [16].

### Droplet digital PCR breakpoint detection assay

#### Primers design

The sequences of BCR-ABL1 genomic junctions (Table 1) were used to design a pair of specific primers for each patient (see Supplementary Table 1) using the Primer3Plus tool (http://www.bioinformatics.nl/cgi-bin/primer3plus/primer3plus.cgi). The primer forward maps at the 5′ and the primer reverse maps at the 3′ of the breakpoint site (60–200 bp product length). Before ddPCR analysis, a qualitative PCR was performed for each pair of primers to verify their specificity, using the Platinum Taq DNA Polymerase (Thermo Fisher Scientific), 200 ng of genomic DNA, in a final volume of 50 μl (thermal-cycling conditions: 95° C for 5 min, 95° C for 30 s, 60° C for 20 s, 72° C for 20 s (35 cycles), 72° C for 8 min and 4° C hold). Each pair of primers was tested on the specific patient, on a NC (a CML patient with a different breakpoint) and a NTC. The PCR products were visualized on an 1.5% agarose-gel. Furthermore, a melting curve assay was performed by qRT-PCR experiments with the LightCycler 480 SYBR Green I Master mix on the LightCycler 480II (Roche Diagnostics, Indianapolis, IN). Amplification thermal protocol: 95° C for 10 min, 95° C for 10 s, 60° C for 30 s, and 72° C for 1 min (45 cycles). The analysis was performed using the LightCycler 480 Software 1.5.1 (Roche Diagnostics).

### EvaGreen assay

The breakpoint detection by ddPCR was conducted by adding to the QX200 ddPCR EvaGreen Supermix (BioRad), the forward primer (200 nM), the reverse primer (100 nM), 10 ng of gDNA (from PB), in a final volume of 20 μl. ddPCR data were analyzed with QuantaSoft analysis software (version 1.7.4). Furthermore, for each sample, the quantification of ZP3 (zona pellucida glycoprotein 3) gene was conducted in a different well (see Supplementary Table 1). The amount of disease was calculated as the percentage ratio between the number of genomes with the BCR-ABL1 fusion gene and the total genomes in the well (ZP3/2).

### TaqMan assay

For case#1 a TM hydrolysis probe was designed according to Droplet Digital PCR guidelines (see supplementary table). The BCR-ABL1 breakpoint detection was conducted, adding to 2X ddPCR Supermix for Probes (no dUTP), the target primers/probe (20X – FAM conjugated), the reference primers/probe (20X – HEX conjugated) specific for the EIF2C1 (eukaryotic translation initiation factor 2C, 1) gene, 50 ng of gDNA, in a final volume of 20 μl. Each reaction (replicated 16 times) was partitioned into 20,000 droplets by the droplet generator and amplified in a C1000 Touch thermal cycler: 95° C for 10 min, 94° C for 30 s, 60° C for 1 min, (40 cycles), 98° C for 10 min and 4° C hold. The amount of disease was calculated as the percentage ratio between the number of genomes with the BCR-ABL1 fusion gene and the total genomes in the well (EIF2C1/2).

## SUPPLEMENTARY MATERIALS TABLE


