# Supplementary material for: Genomic BCR-ABL1 breakpoint characterization by a multi-strategy approach for “personalized monitoring” of residual disease in chronic myeloid leukemia patients
Source: Oncotarget. 2018 Jan 5;9(13):10978–86. doi: 10.18632/oncotarget.23971 (PMC5834283; doi:10.18632/oncotarget.23971)
Supplement: Supplementary file 1 [file oncotarget-09-10978-s001.pdf]

Genomic BCR-ABL1 breakpoint characterization by a multi-strategy approach for "personalized monitoring" of residual disease in chronic myeloid leukemia patients

SUPPLEMENTARY MATERIALS

Supplementary Table 1: Sequences of primers used for SS, for ddPCR EG assays and for ddPCR TM assay

	Name	Sequence (5′->3′)
Nested primers for SS	BCRex13_seq	CATTCCGCTGACCATCAATA
	BCRint13-14_291F	ATGCTCTGTGCCTTGGATCT
	BCRex14_seq	GTCCACTCAGCCACTGGATT
	BCRint14-15_3F	AAGTACTGGTTTGGGGAGGA
	BCRint14-15_278F	TCGAGTAATTGCAGGGGTTT
	BCRint14-15_764F	AGCTTGTGCCACTGCATTC
	BCRint14-15_1278F	CATCCCCAAACCAAACCTATT
	BCRint14-15_1748F	ATTGTAGGGGCTTCCCACAT
Case#1 (EG assay)	Cs#1F	CCTTGCCTCCCTGTTACCTT
	Cs#1R	CCCAGGTAGCTGGTACTACA
Case#2 (EG assay)	Cs#2F	TAGCCTGTCTCAGATCCTGG
	Cs#2R	GAGGGAGAGGGGGAAGTATT
Case#3 (EG assay)	Cs#3F	GCACGGCTTCTGTTCCTAGT
	Cs#3R	TGCTGCTTCCCCACTTGATT
Case#4 (EG assay)	Cs#4F	GGCAAGGACTTTGACAGACA
	Cs#4R	TCCTCACGCATTCTTTCCTG
Case#5 (EG assay)	Cs#5F	CTTCGAGTCACTGGTTTGCC
	Cs#5R	TCTCTCTCTCTCTCAAGAACCT
Case#6 (EG assay)	Cs#6F	GGCACCTGTAATCACAACTGC
	Cs#6R	TCAAGTAAAAATGGCAGTCCA
Case#7 (EG assay)	Cs#7F	CCTTCTGGGTGTGGAATTGT
	Cs#7R	TTCACTGGATCTGTGCATGA
Case#8 (EG assay)	Cs#8F	GGAGTCCCCTTTGCCTTAAC
	Cs#8R	CGCCTGTAGTACCCTCTACT
Case#9 (EG assay)	Cs#9F	TGACACTGGCTTACCTTGTG
	Cs#9R	CTACATACAGGCTTGTGCCA
Case#10 (EG assay)	Cs#10F	CACAGCATTCCGCTGACCATCA
	Cs#10R	CCTGGTGGCTCCAGTTTTCTAC
Reference gene (EG assay)	ZP3F	CATCCTGAGACGTCCGTACA
	ZP3R	CTGACCACATCTTCTGTGTCC
Case#1 (TM assay)	Cs#1prF	CATGACATGCAGATTGCACC
	Cs#1prR	CCCAGGTAGCTGGTACTACA
	Cs#1probe	TGCCCCGTGGTCCCGGGCTT
